# A Possible Link between Gastric Mucosal Atrophy and Gastric Cancer after *Helicobacter pylori* Eradication

**DOI:** 10.1371/journal.pone.0163700

**Published:** 2016-10-05

**Authors:** Tomomitsu Tahara, Tomoyuki Shibata, Noriyuki Horiguchi, Tomohiko Kawamura, Masaaki Okubo, Takamitsu Ishizuka, Mitsuo Nagasaka, Yoshihito Nakagawa, Naoki Ohmiya

**Affiliations:** Department of Gastroenterology, Fujita Health University School of Medicine, Toyoake, Japan; University Hospital Llandough, UNITED KINGDOM

## Abstract

**Background:**

The effect of *H*. *pylori* eradication in gastric cancer prevention can be attributed to the improvement of atrophic gastritis, which is a known risk of gastric cancer. However, gastric cancer has also been diagnosed after long-term *H*. *pylori* eradication. This study aimed to clarify the association between gastric atrophy and gastric cancer after *H*. *pylori* eradication, including its clinicopathological features.

**Methods:**

A total of 55 consecutive patients with 64 early gastric cancers (EGCs) diagnosed after *H*. *pylori* eradication were enrolled. The degree of endoscopic atrophy and the histological degrees of mononuclear cell infiltration, atrophy, and metaplasia in the corpus and adjacent mucosa of the EGCs were determined and scored.

**Results:**

The majority of EGCs (63/64) were located within the endoscopically assessed atrophic mucosa or along the atrophic border. The adjacent mucosa of the EGCs presented significantly higher degrees of all histological parameters than in the corpus (mononuclear cell infiltration, 0.86+/-0.09 *vs*. 0.51+/-0.11, *P* = 0.016; atrophy, 1.77+/-0.13 *vs*. 0.65+/-0.14, *P*<0.0001; metaplasia, 1.68+/-0.13 *vs*. 0.48+/-0.1, *P*<0.0001). The degree of endoscopic atrophy improved in the patients with longer post-*H*. *pylori* eradication periods; however, this trend was not observed for the histological parameters, and high degrees of atrophy and metaplasia were observed in the adjacent mucosa of the EGCs compared with the corpus during all periods (all *P*<0.05). The histological degrees of atrophy and metaplasia in the adjacent mucosa were particularly higher in the patients who underwent eradication due to gastric ulcers.

**Conclusions:**

Severe gastric atrophy remained in the adjacent mucosa of the EGCs after *H*. *pylori* eradication, which may be linked to gastric carcinogenesis.

## Introduction

Helicobacter pylori (*H*. *pylori*) infection is a known risk factor of gastric cancer development, according to epidemiological and experimental studies [1–5]. It has been demonstrated that *H*. *pylori* eradication significantly reduces the incidence of metachronous gastric cancer following endoscopic resection of early gastric cancer [6]. Therefore, the Japanese national health insurance system has covered the cost of eradication therapy for all patients with *H*. *pylori*-associated gastritis.

However, after successful *H*. *pylori* eradication, gastric cancers are sometimes identified [7]. These cancers have some characteristic features, such as tiny and flattened lesions [8], which are difficult to diagnose endoscopically. Identifying those patients who are at high risk of developing gastric cancer after *H*. *pylori* eradication is important to ensure better clinical implementation.

It is well known that the pathological state of gastritis is closely associated with the risk of gastric cancer among patients with *H*. *pylori*. For example, severe gastric mucosal atrophy or intestinal metaplasia are strong risk factors for gastric cancer, particularly the differentiated (intestinal) phenotype [3]. The effect of *H*. *pylori* eradication in preventing gastric cancer can, therefore, be attributed to the improvement of atrophic gastritis. Indeed, the patients with baseline severe atrophic gastritis have an increased risk of gastric cancer after *H*. *pylori* eradication because gastric atrophy does not improve in the short term [7]. However, gastric cancers may also be identified after long-term *H*. *pylori* eradication (e.g., after ten years). The association between gastric atrophy and gastric cancer risk is unclear for such patients. Therefore, this study was designed to clarify the status of atrophic gastritis at the time point that patients developed gastric cancer after *H*. *pylori* eradication.

Through the analysis of macroscopic and histological levels and the association with clinicopathological features, we determined the degree of atrophic gastritis in the gastric corpus and adjacent mucosa of patients with early gastric cancer (EGC) that was diagnosed after *H*. *pylori* eradication.

## Materials and Methods

### Ethics statement

This study was approved by the Human Research Ethics Committee of the Fujita Health University School of Medicine. Each participant provided written informed consent for their clinical and laboratory data to be used and published for research purposes. The study was conducted according to the principles expressed in the Declaration of Helsinki.

### Subjects

We enrolled 55 consecutive patients with 64 EGCs that were diagnosed after successful *H*. *pylori* eradication therapy between April 2007 and September 2015. Four lesions from three patients were diagnosed as EGC 6 months after successful *H*. *pylori* eradication, while the remaining 60 lesions were diagnosed as EGC at least 12 months after successful *H*. *pylori* eradication. For 24 patients (43.6%), the post-*H*. *pylori* eradication period was less than 3 years; the period was 3 to 9 years for 21 patients (38.2%) and more than 10 years for 10 patients (18.2%) (Table 1). All patients had history of successful *H*. *pylori* eradication therapy and we also evaluated the *H*. *pylori* status at the time point of the enrollment by the urea breath test as well as histological assessments using endoscopic biopsy specimens obtained from non-pathological mucosa of the greater curvature of the gastric antrum and upper corpus. Since the results were negative for both examinations for all patients, we considered that *H*. *pylori* eradication had been successfully performed. Some of these patients were recruited from our recent study investigating the clinicopathological features of EGC after *H*. *pylori* eradication (Horiguchi et al, submitted). The reasons for the *H*. *pylori* eradication were peptic ulcer disease (n = 19), chronic gastritis (n = 24) or endoscopic resection of EGC (n = 12). All patients attended the Endoscopy Center of Fujita Health University for endoscopic submucosal dissection (ESD) between April 2007 and September 2015. ESD was performed for 53 lesions, while laparoscopy-assisted gastrectomy, which was considered to be the surgical indication during the initial endoscopic examination, was performed for 11 lesions. The clinicopathological subtypes of the EGCs, including the anatomic location, morphologic appearance, depth, color, histologic type and tumor size, were investigated based on the patients’ medical records. Based on the endoscopic pictures, an indistinct case was defined if the lateral extension was indistinguishable through the entire marginal area. This judgment was made through consensus by two expert endoscopists (NH and TT).

**Table 1:** The clinicopathological characteristics of 64 EGCs from 55 patients diagnosed after *H*. *pylori* eradication.

Variables	
Median age (range)	72 (53–89)
Males (n)	70.9% (39)
Reason for *H*. *pylori* eradication: post ESD/GU/other (n)	21.8%/34.6%/43.6%(12/19/24)
Duration after *H*. *pylori* eradication: 3 y</3-9 y/10 y~ (n)	43.6%/38.2%/18.2% (24/21/10)
Location: U/M/L (n)	18.8%/40.6%/40.6% (12/26/26)
Degree of endoscopic atrophy: C1/C2/C3/O1/O2/O3 (n)	9.1%/18.2%/25.5%/29.1%/7.3%/10.9% (5/10/14/16/4/6)
Morphology: elevated/depressed (n)	14.1%/85.9% (9/55)
Color: Redness/Whiteness/same as surroundings (n)	82.8%/12.5%/4.7% (53/8/3)
Histology: differentiated/undifferentiated (n)	95.3%/4.7% (61/3)
Tumor size: ±SE	15.2± 1.4 mm
Depth: M/SM< (n)	84.4%/15.6% (54/10)
Treatment: ESD/ gastrectomy after ESD/gastrectomy (n)	78.1%/4.7%/17.2% (50/3/11)
Indistinct case (n)	18.8% (12)

GU, gastric ulcer; U, upper; M, middle; L lower; M, intra-mucosal cancer; SM, submucosa

### Evaluation of gastric mucosal atrophy

The extent of the endoscopically assessed gastric atrophy was evaluated using the Kimura-Takemoto classification system and then scored from normal to C1–C3 and from O1–O3. C and O indicate closed-type and open-type gastritis, respectively. The atrophic area is localized to the antral region in C-1, and the area expands in C2, C3, O1, O2, and O3. O3 corresponds to panatrophic gastritis [9]. We scored C1-C3 and O1-O3 as 1–3 and 4–6, respectively and cases who have no atrophy were considered as score 0.

Using biopsy specimens from the uninvolved mucosa of the greater curvature of the gastric corpus, the extent of mononuclear cell infiltration, atrophy, and metaplasia was assessed histologically using the updated Sydney system [10], with each factor scored from 0 (normal) to 3 (marked). This assessment was also performed for the adjacent mucosa of the EGC (within 1 cm) using the resected specimens.

### Statistical analysis

Continuous variables between the two groups were determined using Student's t-test. Categorical variables were determined using the Chi-squared test, and P<0.05 was considered to be statistically significant.

## Results

### Clinicopathological characteristics of the EGCs

The clinicopathological characteristics of 64 EGCs from 55 patients are shown in Table 1. The majority of the EGCs were depressed (85.9%) and reddish (82.8%) lesions as well as histologically differentiated adenocarcinoma (95.3%). Ten lesions had invaded the submucosa (SM) or deeper layers; one of these lesions had invaded into the muscularis propria, and the remaining lesions had invaded the SM.

### Possible link between gastric mucosal atrophy and gastric cancer after H. pylori eradication

**Fig 1:**
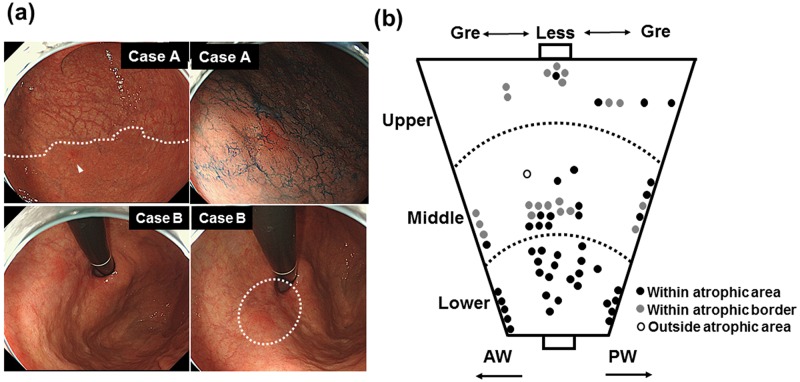
The possible link between gastric mucosal atrophy and early gastric cancer (EGC) after *H*. *pylori* eradication. (a) Two typical types of EGC, in relation to the endoscopically assessed atrophic mucosa. In case A, a cancerous lesion was observed as the reddish area (white arrowhead) within the atrophic border (white dotted line). In case B, a cancerous lesion was observed as the reddish area (white dotted line) within the atrophic area. (b) The association between the endoscopically assessed atrophic mucosa and the locations of 64 EGC lesions. The black circle shows the EGCs located within atrophic area; the grey circle shows the EGCs located within atrophic border; the white circle shows the EGCs located outside the atrophic area; gre, greater curvature; less, lesser curvature; AW, anterior; PW posterior.

**Fig 2:**
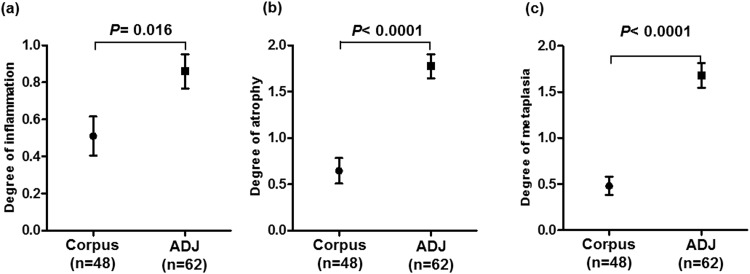
The histological degrees of mononuclear cell infiltration, atrophy, and metaplasia in the corpus and adjacent (ADJ) mucosa in the EGC patients. Each factor was scored from 0 (normal) to 3 (marked). The statistical analysis was performed using Student's t-test.

**Fig 3:**
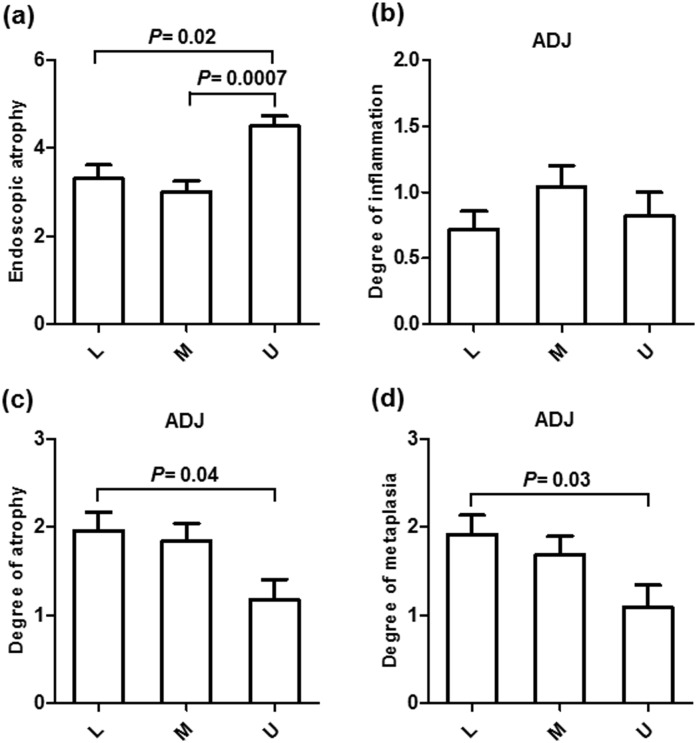
The degree of endoscopic atrophy, histological degrees of mononuclear cell infiltration, atrophy, and metaplasia in the corpus and adjacent (ADJ) mucosa compared to the anatomical location of gastric cancer. The statistical analysis was performed using Student's t-test. L, lower; M, middle; U, upper.

To investigate the potential link between gastric atrophy and gastric cancer after *H*. *pylori* eradication, we initially checked the association between the extensions of endoscopically atrophic mucosa and the locations of the EGC lesions (Fig 1). Among the 64 lesions, 43 lesions (67.2%) were located within the atrophic area, and 20 lesions (31.3%) were located within the atrophic border. Only one lesion (1.6%) was located outside the atrophic area, suggesting the importance of atrophic mucosa in the development of gastric cancer after *H*. *pylori* eradication. Next, we investigated the histological degrees of mononuclear cell infiltration, atrophy, and metaplasia in the greater curvature of the gastric corpus (n = 48) as well as the adjacent mucosa of the EGC (within 1 cm, n = 62, Fig 2). Because all patients had a prior history of successful *H*. *pylori* eradication, the degrees of all parameters were mild in the corpus. However, the adjacent mucosa of the EGC presented considerable degrees of all parameters (mononuclear cell infiltration, 0.51+/-0.11 *vs*. 0.86+/-0.09, *P* = 0.016; atrophy, 0.65+/-0.14 *vs*. 1.77+/-0.13, *P*<0.0001; metaplasia, 0.48+/-0.1 *vs*. 1.68+/-0.13, *P*<0.0001). The degree of endoscopic atrophy was significantly higher in the patients with EGC located in upper anatomical locations (Fig 3a), while the histological degrees of atrophy and metaplasia in the adjacent mucosa were rated lower in this group compared to patients with EGC in lower anatomical locations (*P* = 0.04 and 0.03, respectively, Fig 3c and 3d).

### Association between gastric mucosal atrophy and gastric cancer after H. pylori eradication, according to various clinicopathological features

**Fig 4:**
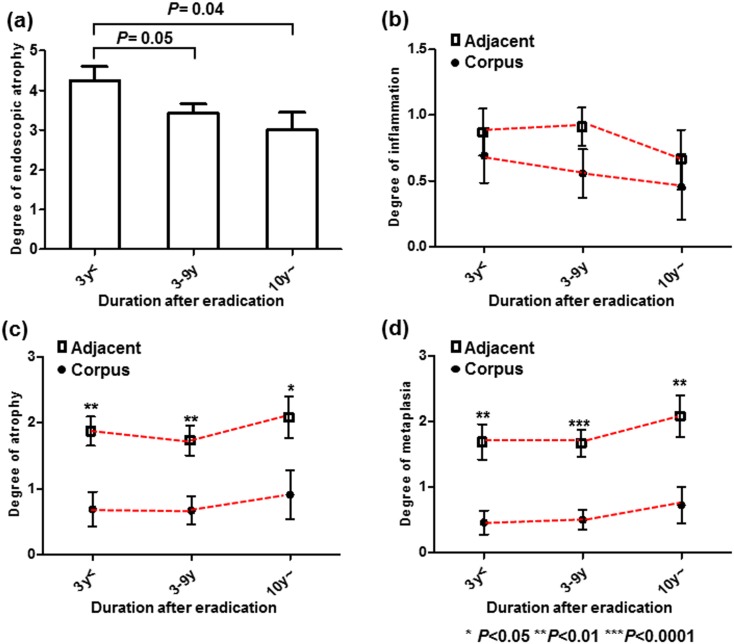
The degree of endoscopic atrophy (a), histological degrees of mononuclear cell infiltration (b), atrophy (c), and metaplasia (d) in the corpus and adjacent mucosa compared to the period after *H*. *pylori* eradication. The statistical analysis was performed using Student's t-test.

**Fig 5:**
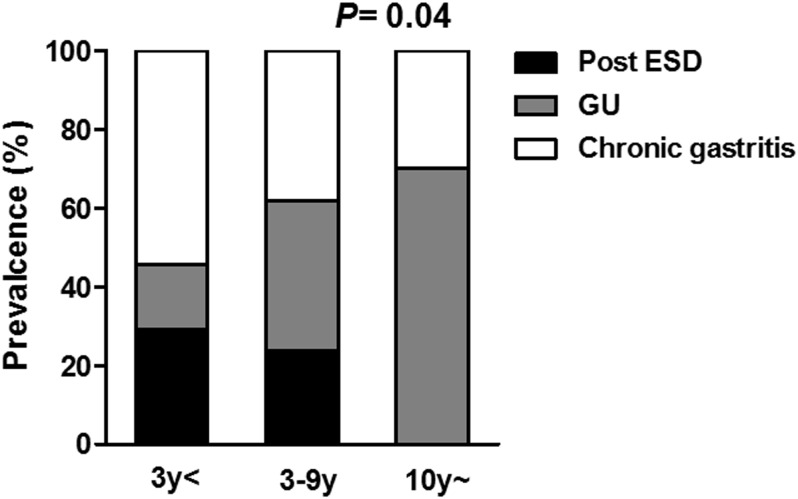
The association between the reasons for *H*. *pylori* eradication and the post-eradication period. Post ESD, patients who underwent eradication after ESD for the gastric cancer; GU, gastric ulcer; the statistical analysis was performed using the chi-squared test.

**Fig 6:**
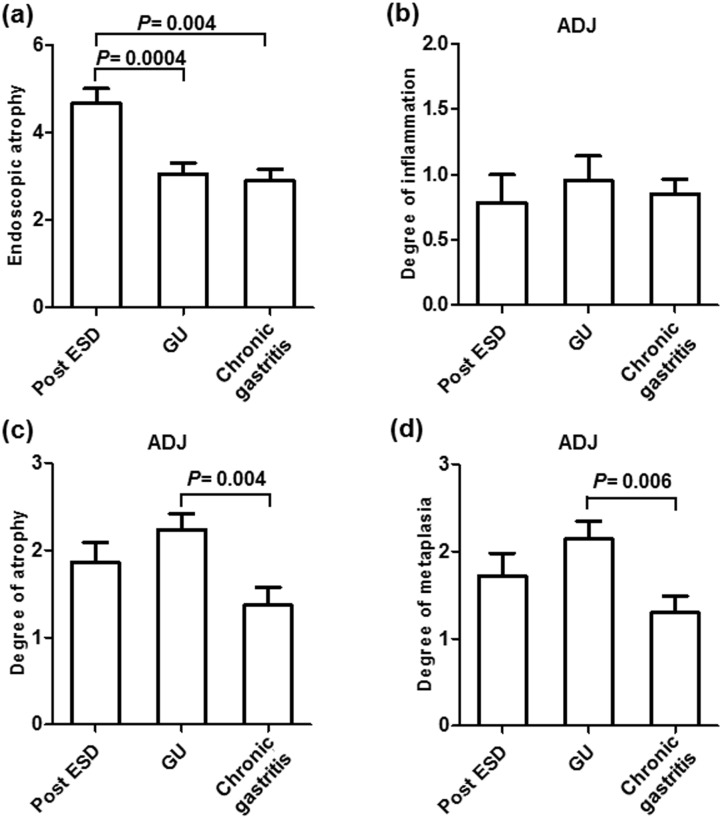
The degree of endoscopic atrophy (a), histological degrees of mononuclear cell infiltration (b), atrophy (c), and metaplasia (d) in the adjacent mucosa (ADJ) compared to the reasons for *H*. *pylori* eradication. Post ESD, patients who underwent eradication after ESD for gastric cancer; GU, gastric ulcer; the statistical analysis was performed using Student's t-test.

We then investigated whether the gastric mucosal atrophy was associated with various clinicopathological features of EGCs after *H*. *pylori* eradication. When dividing the cases according to the post-eradication period, the degree of endoscopic atrophy improved in patients with longer period post-*H*. *pylori* eradication periods (Fig 4a). However, this trend was not observed for all histological parameters, including the corpus and adjacent mucosa (all *P*>0.1), and higher degrees of atrophy and metaplasia were observed in the adjacent mucosa of the EGCs compared to the corpus for all periods (all *P*<0.05, Fig 4c and 4d). Regarding the reasons for *H*. *pylori* eradication and the duration after eradication, the number of patients who underwent eradication after ESD decreased as the duration increased, while the number of patients who underwent eradication due to gastric ulcers considerably increased (*P*<0.05, Fig 5). As expected, the degree of endoscopic gastric atrophy in patients who underwent eradication after ESD was higher compared to the other patients (Fig 6a). However, the histological degrees of atrophy and metaplasia in the adjacent mucosa were particularly higher in the patients who underwent eradication due to gastric ulcers, and this association was significant compared to the patients who underwent eradication due to chronic gastritis (*P* = 0.004, 0.006, respectively, Fig 6c and 6d). This finding suggests that patients who underwent eradication due to gastric ulcers have more severe atrophy and metaplasia in the adjacent mucosa, despite their longer post-eradication periods.

Several studies have reported that gastric cancer after successful *H*. *pylori* eradication provides distinct histological features, such as normal epithelium and/or surface differentiation covering the tumor tissue, which sometimes makes an endoscopic diagnosis difficult [8, 11–13]. In this study, 12 lesions were considered to be indistinct cases because their lateral extensions were endoscopically undistinguishable through the entire margin area. We found that the histological degrees of atrophy and intestinal metaplasia were significantly higher in such cases compared to other cases, particularly in the corpus (*P* = 0.01, 0.02, respectively, S1d and S1e Fig). Although such trends were not observed in the adjacent mucosa, the degree of mononuclear cell infiltration was also higher in the same cases in the adjacent mucosa (P<0.05, S1f Fig).

We also investigated the association between endoscopic and histological atrophy and other clinicopathological features of EGC (S1 Table). Several clinicopathological features were correlated with endoscopic and histological atrophy. The male patients were associated with more severe endoscopic atrophy, histological atrophy and metaplasia in the adjacent mucosa (*P* = 0.05, 0.015, 0.014, respectively). The same associations were found for the differentiated histological type (*P* = 0.017, 0.001, 0.004, respectively). However, lesions measuring more than 20 mm in size were associated with more severe mononuclear cell infiltration, atrophy, and metaplasia in the corpus (*P* = 0.013, 0.007, and 0.012, respectively).

## Discussion

We showed that the majority of EGC lesions (63/64) were located in the endoscopic atrophic mucosa or at the atrophic border. Histological assessment also confirmed that severe atrophy and intestinal metaplasia were observed in the adjacent mucosa of EGCs compared to the corpus, indicating that severe gastric mucosal atrophy is risk factors for developing gastric cancer after *H*. *pylori* eradication.

Several studies have demonstrated that the patients with baseline severe atrophic gastritis have an increased risk of gastric cancer after *H*. *pylori* eradication because the gastric atrophy did not improve in the short term [7,11,12], while there have been few information regarding the gastric atrophy at the time point that the patients developed gastric cancer after eradication. We showed that extension of endoscopic atrophy gradually decreased in patients with longer period post-*H*. *pylori* eradication periods. On the other hand, the histological degrees of atrophy and intestinal metaplasia in the adjacent mucosa were always higher in such patients. This raises the possibility that the gastric cancer patients with longer post-eradication periods might have had more severe baseline severe atrophic gastritis prior to the eradication. In the same time, the result also indicates that it would be important to pay attention to the remaining atrophic area even if its extent is mild to screen gastric cancer especially in patients with longer post-eradication period.

We have shown that the patients with EGC located in the upper anatomical location showed more severe endoscopic gastric atrophy, while the histological degrees of atrophy and intestinal metaplasia were milder in the same patients. This paradoxical phenomenon can be explained by the natural history of *H*. *pylori*-related gastritis. Gastric atrophy caused by *H pylori* infection usually starts from the antrum and then extends to the gastric corpus. It is reasonable to expect that the degrees of atrophy and intestinal metaplasia are milder in the corpus compared to the antrum. However, note that patients with severe endoscopic atrophy in the corpus may have a risk of developing gastric cancer in upper anatomical locations, even if their histological atrophy and intestinal metaplasia are mild. This issue may be important when monitoring for gastric cancer after *H*. *pylori* eradication.

Regarding the reasons for *H*. *pylori* eradication and its duration after eradication, the number of patients who underwent eradication after ESD decreased as the duration time increased, while the number of patients who underwent eradication due to gastric ulcer considerably increased. As expected, the degree of endoscopic gastric atrophy in patients who underwent eradication after ESD was higher compared to that in the other patients, and we found that the patients who underwent eradication due to gastric ulcer presented with a high degree of histological atrophy and intestinal metaplasia in the adjacent mucosa compared to the other patients. This result indicates that the patients who previously had gastric ulcers would have more severe levels of histological atrophy and intestinal metaplasia, which were linked to the risk of gastric cancer, even after long-term *H*. *pylori* eradication. Gastritis patients with gastric ulcers are characterized by more severe damage to the surface epithelium, which may lead to multifocal atrophic gastritis. Therefore, patients with gastric ulcers are considered to be at high risk of developing gastric cancer [13–15]. This concept would also be applicable for patients following *H*. *pylori* eradication. Thus, scheduled endoscopic surveillance should be performed for gastric ulcer patients after *H*. *pylori* eradication.

Several reports have suggested that gastric cancer after successful *H*. *pylori* eradication demonstrates distinct histological features, such as normal epithelium and/or surface differentiation covering the tumor tissue [16–18], which sometimes make an endoscopic diagnosis difficult [17,18]. In this study, 12 EGC lesions were considered to be indistinct cases because their lateral extensions were endoscopically undistinguishable through the entire marginal area. We have shown that indistinct cases had significantly higher degrees of histological atrophy and intestinal metaplasia in the corpus but not in the adjacent mucosa. In these cases, interspersed regenerated gastric crypts were observed in the remaining gastric atrophy in the gastric corpus (data not shown). It may be speculated that such histological conditions in the gastric corpus may be associated with indistinct cases of EGC after *H*. *pylori* eradication.

In summary, we showed that the majority of gastric cancer after *H*. *pylori* eradication develop in the endoscopic atrophic mucosa or at the atrophic border, which is characterized as high degrees of histological atrophy and intestinal metaplasia. Therefore, the endoscopic surveillance of gastric cancer after *H*. *pylori* eradication should be performed, always paying the careful attention to the atrophic area. We should also consider patients who undergo endoscopic resection of gastric cancer as well as patients with a history of peptic ulcers, who are at risk for developing cancer after *H*. *pylori* eradication. Our findings also deserve more intensive study to establish the strategy for the early diagnosis, treatment, and prevention of gastric cancer after *H*. *pylori* eradication.

## Supporting Information

S1 Fig A representative endoscopic picture of indistinct case (a). The cancerous lesion was seen within the yellowish dotted line but its lateral extension was unclear. Degree of endoscopic atrophy (b), histological degrees of mononuclear cell infiltration, atrophy, and metaplasia in the corpus (c, d and e, respectively) and adjacent mucosa (f, g and h, respectively) in relation to the indistinct case. Statistical analysis was performed by the Student's t-test.
(TIF)

S1 Table Association between endoscopic atrophy and histological parameters with subtypes of EGC after H. pylori eradication. (DOCX)
