# Supplementary figures and images for: A Possible Link between Gastric Mucosal Atrophy and Gastric Cancer after Helicobacter pylori Eradication
Source: PLoS One. 2016 Oct 5;11(10):e0163700. doi: 10.1371/journal.pone.0163700 (PMC5051933; doi:10.1371/journal.pone.0163700)

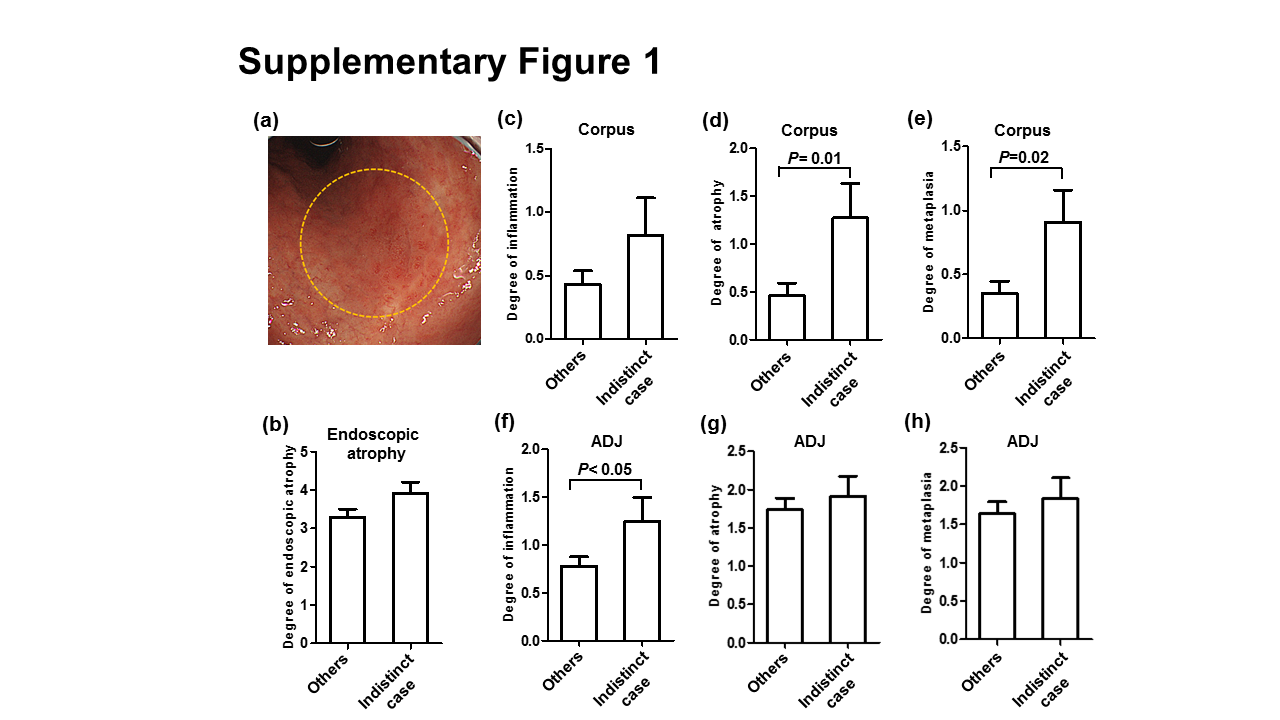

Supplement: S1 Fig — A representative endoscopic picture of indistinct case (a). The cancerous lesion was seen within the yellowish dotted line but its lateral extension was unclear. Degree of endoscopic atrophy (b), histological degrees of mononuclear cell infiltration, atrophy, and metaplasia in the corpus (c, d and e, respectively) and adjacent mucosa (f, g and h, respectively) in relation to the indistinct case. Statistical analysis was performed by the Student's t-test. (TIF) [file pone.0163700.s001.tif]
