# Supplementary material for: A Possible Link between Gastric Mucosal Atrophy and Gastric Cancer after Helicobacter pylori Eradication
Source: PLoS One. 2016 Oct 5;11(10):e0163700. doi: 10.1371/journal.pone.0163700 (PMC5051933; doi:10.1371/journal.pone.0163700)
Supplement: S1 Table — (DOCX) [file pone.0163700.s002.docx]

**Supplementary Table 1** Association between endoscopic atrophy and histological parameters with subtypes of EGC after H. pylori eradication

| Variables | Endoscopic | Inflammation | Atrophy | Metaplasia | Inflammation | Atrophy | Metaplasia |
| --- | --- | --- | --- | --- | --- | --- | --- |
|  | atrophy | (corpus) | (corpus) | (corpus) | (ADJ) | (ADJ) | (ADJ) |
| *Age* |  |  |  |  |  |  |  |
| 75< | 3.20+/-0.26 | 0.50+/-0.14 | 0.64+/-0.18 | 0.46+/-0.13 | 0.80+/-0.13 | 1.80+/-0.18 | 1.71+/-0.20 |
| 75~ | 3.66+/-0.24 | 0.55+/-0.17 | 0.65+/-0.22 | 0.50+/-0.15 | 0.96+/-0.14 | 1.74+/-0.18 | 1.63+/-0.18 |
| *Gender***#** |  |  |  |  |  |  |  |
| Male | **3.62+/-0.21** | 0.55+/-0.12 | 0.68+/-0.16 | 0.50+/-0.11 | 0.91+/-0.11 | **1.96+/-0.15** | **1.87+/-0.16** |
| Female | **2.82+/-0.31** | 0.40+/-0.22 | 0.50+/-0.31 | 0.40+/-0.22 | 0.75+/-0.19 | **1.25+/-0.23** | **1.13+/-0.20** |
| *Morphology* |  |  |  |  |  |  |  |
| Elevated | 3.33+/-0.65 | 0.50+/-0.22 | 0.50+/-0.22 | 0.50+/-0.22 | 1.11+/-0.26 | 1.78+/-0.36 | 1.67+/-0.33 |
| Depressed | 3.42+/-0.19 | 0.52+/-0.12 | 0.67+/-0.16 | 0.48+/-0.11 | 0.83+/-0.10 | 1.77+/-0.14 | 1.68+/-0.15 |
| *Color*: |  |  |  |  |  |  |  |
| Redness | 3.40+/-0.21 | 0.50+/-0.11 | 0.55+/-0.14 | 0.43+/-0.10 | 0.81+/-0.10 | 1.75+/-0.14 | 1.64+/-0.15 |
| Whiteness | 3.50+/-0.27 | 0.50+/-0.34 | 1.00+/-0.45 | 0.67+/-0.33 | 1.29+/-0.29 | 2.14+/-0.34 | 2.14+/-0.34 |
| Same as surroundings | 3.33+/-0.33 | 1.00+/-1.00 | 1.50+/-1.50 | 1.00+/-1.00 | 1.00+/-0.00 | 1.33+/-0.67 | 1.33+/-0.67 |
| *Histology***$** |  |  |  |  |  |  |  |
| Differentiated | **3.52+/-0.18** | 0.57+/-0.11 | 0.71+/-0.15 | 0.52+/-0.11 | 0.91+/-0.10 | **1.88+/-0.13** | **1.78+/-0.13** |
| Undifferentiated | **1.75+/-0.75** | 0.00+/-0.00 | 0.00+/-0.00 | 0.00+/-0.00 | 0.25+/-0.25 | **0.25+/-0.25** | **0.25+/-0.25** |
| *Tumor size***&** |  |  |  |  |  |  |  |
| <20 | 3.37+/-0.21 | **0.35+/-0.10** | **0.41+/-0.11** | **0.32+/-0.09** | 0.79+/-0.11 | 1.79+/-0.16 | 1.66+/-0.16 |
| 20~ | 3.53+/-0.34 | **0.93+/-0.25** | **1.21+/-0.35** | **0.86+/-0.23** | 1.13+/-0.17 | 1.73+/-0.23 | 1.73+/-0.23 |
| *Depth* |  |  |  |  |  |  |  |
| M | 3.35+/-0.20 | 0.53+/-0.11 | 0.65+/-0.15 | 0.50+/-0.11 | 0.89+/-0.11 | 1.85+/-0.13 | 1.75+/-0.14 |
| SM< | 3.70+/-0.45 | 0.5+/-0.33 | 0.63+/-0.42 | 0.38+/-0.26 | 0.80+/-0.20 | 1.40+/-0.40 | 1.30+/-0.42 |
| All data were expressed as mean +/- SEM. Statistical analysis was performed by the Student's t-test. ADJ, adjacent; | | | | | | |  |
| Bold text indicates a statistically significant relationship. | | |  |  |  |  |  |
| **#** Endoscopic atrophy: *P*=0.05, Atrophy (ADJ): *P*=0.015, Metaplasia (ADJ): *P*=0.014 | | | | |  |  |  |
| **$** Endoscopic atrophy: *P*=0.017, Atrophy (ADJ): *P*=0.001, Metaplasia (ADJ): *P*=0.004 | | | | |  |  |  |
| **&** Inflammation (corpus): *P*=0.013, Atrophy (corpus): *P*=0.007, Metaplasia (corpus): *P*=0.012. | | | | | |  |  |
